# Compilation of longitudinal microbiota data and hospitalome from hematopoietic cell transplantation patients

**DOI:** 10.1038/s41597-021-00860-8

**Published:** 2021-03-02

**Authors:** Chen Liao, Bradford P. Taylor, Camilla Ceccarani, Emily Fontana, Luigi A. Amoretti, Roberta J. Wright, Antonio L. C. Gomes, Jonathan U. Peled, Ying Taur, Miguel-Angel Perales, Marcel R. M. van den Brink, Eric Littmann, Eric G. Pamer, Jonas Schluter, Joao B. Xavier

**Affiliations:** 1 Program for Computational and Systems Biology, Memorial Sloan-Kettering Cancer Center, New York, NY USA; 2 Department of Health Sciences, Università degli Studi di Milano, Milan, Italy; 3 Infectious Disease Service, Department of Medicine, and Immunology Program, Sloan Kettering Institute, New York, NY USA; 4 Department of Immunology, Memorial Sloan-Kettering Cancer Center, New York, NY USA; 5 Adult Bone Marrow Transplantation Service, Department of Medicine, Memorial Sloan Kettering Cancer Center, New York, NY USA; 6 Weill Cornell Medical College, New York, NY USA; 7 Duchossois Family Institute, University of Chicago, Chicago, IL USA

**Keywords:** Cancer, Databases, Time series, Microbiome, Data mining

## Abstract

The impact of the gut microbiota in human health is affected by several factors including its composition, drug administrations, therapeutic interventions and underlying diseases. Unfortunately, many human microbiota datasets available publicly were collected to study the impact of single variables, and typically consist of outpatients in cross-sectional studies, have small sample numbers and/or lack metadata to account for confounders. These limitations can complicate reusing the data for questions outside their original focus. Here, we provide comprehensive longitudinal patient dataset that overcomes those limitations: a collection of fecal microbiota compositions (>10,000 microbiota samples from >1,000 patients) and a rich description of the “hospitalome” experienced by the hosts, i.e., their drug exposures and other metadata from patients with cancer, hospitalized to receive allogeneic hematopoietic cell transplantation (allo-HCT) at a large cancer center in the United States. We present five examples of how to apply these data to address clinical and scientific questions on host-associated microbial communities.

## Abstract (summary)

**Table:** 

Measurement(s)	gut microbiome measurement • Cell Density • Clinical Data
Technology Type(s)	DNA sequencing • quantitative PCR • clinical laboratory measurement • 16s ribosomal gene sequencing assay
Factor Type(s)	relative abundance of bacteria • timepoint of hematopoietic cell transplantation • type of hematologic malignancy • time and route of drug administration • type of bacterial infection
Sample Characteristic - Organism	Homo sapiens

Machine-accessible metadata file describing the reported data: 10.6084/m9.figshare.13584986

## Background & Summary

The intestinal microbiota is critical to many aspects of human health^1^, yet most of our knowledge of mechanisms linking microbiota and host phenotypes comes from animal models^2^. Experiments with animals are important, but the specific mechanisms can be hard to translate into human therapies: the gut microbiota can differ between the animal and humans, and so can environmental factors such as the pathogen-free environment, diets, inbreeding, and subject geno- and pheno-types that drive a disease^2^. Instead, associations inferred directly from patients undergoing well-defined perturbations could accelerate the discovery of new mechanisms of microbiota-host interaction to benefit human health^3^. This idea fits the concept of reverse translational research^4^: Microbiota data acquired from well-monitored patients could be reused in other studies beyond the study for which it was originally collected; the longitudinal data could empower causal inference and lead to hypotheses that are more likely to produce microbiota-targeted therapies for patients.

The composition of the gut microbiota measured from feces using 16S rRNA amplicon sequencing can vary widely from person to person and can even change over time within a single person^5–7^. This large variability hampers the inference of microbiota-host associations. Furthermore, many microbiota studies in humans are cross-sectional, have low sample numbers or collect data from outpatients that may lack information on confounding factors such as the status of the immune system of the human host and drugs such as antibiotics. Limitations such as these can introduce statistical bias in microbiome research^8^. Therefore, we recently compiled an extensive hospitalome of our patients, including a vast collection of medication administrations and blood phenotype data^3^. Time-series data of hospitalized patients that link the past events to the future events in an individual person are valuable and necessary assets to reveal “mathematically causal” relationships^9^.

Here we provide a description of the gut microbiota data that we acquired from patients hospitalized to receive allogeneic hematopoietic cell transplantation (allo-HCT) at Memorial Sloan Kettering (MSK). Over the years we have been depositing the raw sequencing data at the NCBI’s short read archive (SRA) in batches called BioProjects. Here we provide links to deposited data for each sample analyzed, which helps the reader obtain a paired-end fastq file for any given sample without having to navigate the multiple BioProjects that our team has submitted over the years. We also provide computer code written in Matlab (The Mathworks Inc., version 2018a), which exemplifies how to analyze and extract insights from the curated data tables that compose this rich longitudinal dataset. We have used these data before in several publications in the past 10 years^3,10–27^, in part or in whole, to design lab experiments with mice that identified mechanisms such as microbiota protection against vancomycin resistant *Enterococcus*^17^, the impact of dietary lactose on the expansion of *Enterococcus* in the gut^20^, the mechanisms of resistance to colonization by *Clostridioides difficile*^19^, and to find microbiome risk factors for Graft-versus-host disease (GVHD)-related mortality^20,21,28^. The data compiled here include >10,000 microbiota samples and clinical metadata from >1,000 patients. We will continue to expand these data as we continue collection, but so far, we include only patients hospitalized for allo-HCT. This choice makes the cohort uniform in crucial ways: The underlying disease is typically a hematologic malignancy such as leukemia or lymphoma, and the patients all have received chemotherapy and in some cases irradiation as conditioning regimen before infusion of hematopoietic cells from a healthy donor to reconstitute the hematopoietic system.

Patients undergoing allo-HCT are carefully monitored during their hospitalizations: HCT is considered the strongest perturbation of the immune system deliberately carried out in humans. Patients receive *prophylactic* antibiotics before the transplant (at MSK during certain years, a combination of a fluoroquinolone and intravenous vancomycin^29^), and often they also receive *empirical* (without evidence of bloodstream infection) or therapeutic (with evidence of bloodstream infection) antibiotics in response to infection symptoms such as a neutropenic fever. Patient treatment regimens and the microbiota dynamics associated with those treatments allowed us to infer the association of individual antibiotic exposures on microbiota composition *in vivo*^10^, and the time-series of white blood cells collected routinely for these patients allowed us to infer the impact of the gut microbiota in the dynamics of the immune system of its human host^3^. But we believe there are many more questions to be addressed.

We compiled this vast longitudinal dataset of microbiota and associated clinical metadata from allo-HCT patients believing that it will interest investigators outside of our center. Here we organize and explain the dataset to aid those investigators in addressing new questions. Our first goal is to compile and annotate easily accessible links to public repositories where the data may be obtained. Our second goal is to guide readers through quantitative analysis of the data. The analysis examples are: displaying a patient’s microbiota timeline and metadata; visualizing the entire dataset using the t-Distributed Stochastic Neighbor Embedding (t-SNE) dimensionality reduction technique^30^ with a patient’s trajectory overlaid on top; quantifying the association between exposure to antibiotics and gut microbiota composition; and performing survival analysis where changes in microbiota composition predict patient risk of bloodstream infection. This compilation of resources facilitates future data access, analysis, and interpretation. In particular, we guide readers through the sample filtering criteria specific to each example.

## Methods

### Human participants and clinical metadata

Sample collection from patients and analysis of the biospecimens were approved by the Memorial Sloan Kettering Cancer Center Institutional Review Board. All participants provided signed informed consent for specimen collection. Clinical metadata, including antibiotics, body temperature, timing and identity of isolates of bloodstream infections were obtained from semi-automated parsing of clinical records with extensive data curation. Absolute blood cell counts were obtained from routine clinical laboratory studies^3^. The data were stripped of all patient identifiable fields such as medical record numbers. The PatientID is a non-identifiable patient number that can be used to link clinical metadata to microbiota sample data. Note that a minority of patients acquired before we systematized our naming convention are named with “patient_witth_sample_####”.

All dates of sample and clinical metadata collection were removed and set to artificial time points. To avoid publication of the calendar dates of events associated with patients, we made the event dates of any patient to be relative to a patient-specific, deidentified reference date (these deidentified dates are provided in columns “Timepoint”, “TimepointOfTransplant”, “StartTimepoint” and “StopTimepoint”; see below). The secret reference dates will not be disclosed. We also provided columns that conveniently represent the event dates relative to the date of nearest HCT of any patient (see below for columns “DayRelativeToNearestHCT”, “StartDayRelativeToNearestHCT”, “StopDayRelativeToNearestHCT”).

### Stool sample extraction, 16S rRNA amplificon sequencing and analysis

A frozen aliquot (≈100 mg) of each fecal sample was suspended, while frozen, in a solution containing 500 μl of extraction buffer [200 mM tris (pH 8.0), 200 mM NaCl, 20 mM EDTA], 200 μl of 20% SDS, 500 μl of phenol/chloroform/isoamyl alcohol (25:24:1), and 500 μl of 0.1-mm-diameter zirconia/silica beads (BioSpec Products). Microbial cells were lysed by mechanical disruption with a bead beater (BioSpec Products) for 2 min, after which two rounds of phenol/chloroform/isoamyl alcohol extraction were performed. DNA was precipitated with ethanol and resuspended in 50 μl of tris/EDTA buffer with ribonuclease (100 μg/ml). The isolated DNA was subjected to additional purification with QIAamp mini spin columns (Qiagen).

For each sample, duplicate 50-μl PCRs were performed, each containing 50 ng of purified DNA, 0.2 mM deoxynucleotide triphosphates, 1.5 mM MgCl2, 2.5 U Platinum Taq DNA polymerase, 2.5 μl of 10 × PCR buffer, and 0.5 μM of each primer designed to amplify the V4-V5: 563 F (5′-nnnnnnnn-NNNNNNNNNNNN-AYTGGGYDTAAAGNG-3′) and 926 R (5′-nnnnnnnn-NNNNNNNNNNNN-CCGTCAATTYHTTTRAGT-3′). A unique 12-base Golay barcode (Ns) precedes the primers for sample identification^31^, and one to eight additional nucleotides were placed in front of the barcode to offset the sequencing of the primers. Cycling conditions were 94 °C for 3 min, followed by 27 cycles of 94 °C for 50 s, 51 °C for 30 s, and 72 °C for 1 min. For the final elongation step, 72 °C for 5 min was used. Replicate PCRs were pooled, and amplicons were purified using the QIAquick PCR Purification Kit (Qiagen). PCR products were quantified and pooled at equimolar amounts before Illumina barcodes and adaptors were ligated, using the Illumina TruSeq Sample Preparation protocol. The completed library was sequenced on an Illumina MiSeq platform following the Illumina recommended procedures with a paired-end 250 × 250 bp kit.

Amplicon sequence variants (ASVs) were identified from 16S paired-end sequencing using the Divisive Amplicon Denoising Algorithm (DADA2) pipeline including filtering and trimming of the reads^32^. Reads were trimmed to remove the first 180 bp or the first point with a quality score Q < 2, and reads containing ambiguous nucleotides (N) or if two or more errors were expected based on the quality of the trimmed read were removed. Taxonomy was assigned to ASVs using a 8-mer based classifier trained by IDTaxa^33^ using the SILVA database^34^.

### Quantification of microbiota density and detection of the *vanA* gene

qPCR was performed on DNA extracted from the samples using DyNAmo SYBR Green qPCR kit (Finnzymes) and 0.2 μM of the universal bacterial primer 8 F (5′-AGAGTTTGATCCTGGCTCAG) and the broad-range bacterial primer 338 R (5′-TGCTGCCTCCCGTAGGAGT-3′). Standard curves were prepared by serial dilution of the PCR blunt vector (Invitrogen) containing 1 copy of the 16 s rRNA gene. Cycling conditions were 95 °C for 10 minutes followed by 40 cycles of 95 °C for 30 seconds, 52 °C for 30 seconds, and 72 °C for 1 minute. Detection of the *vanA* gene was conducted via PCR performed using specific primers for the *vanA* gene: forward, 5′-AATCGGCAAGACAATATGAC; reverse, 5′-ACCTCGCCAACAACTAACGC.

## Data Records

**Fig. 1:**
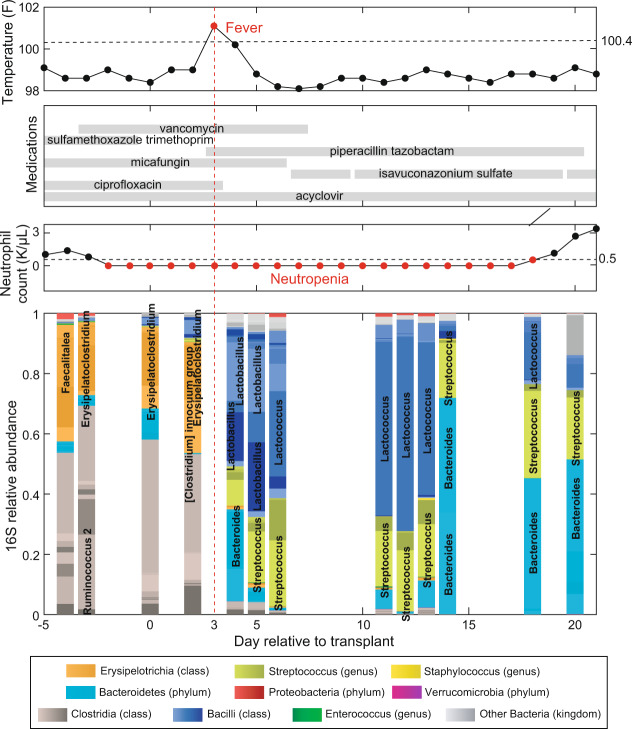
Timeline of clinical events and microbiota composition for a representative patient (PatientID = 1511) receiving hematopoietic cell transplantation at MSK. Day 0 is the day the patient received the infusion of hematopoietic cells (day of HCT). Negative days represent pre-transplant days and positive days represent post-transplant days. The data shown here are for the period from day −5 to day +21.

**Fig. 2:**
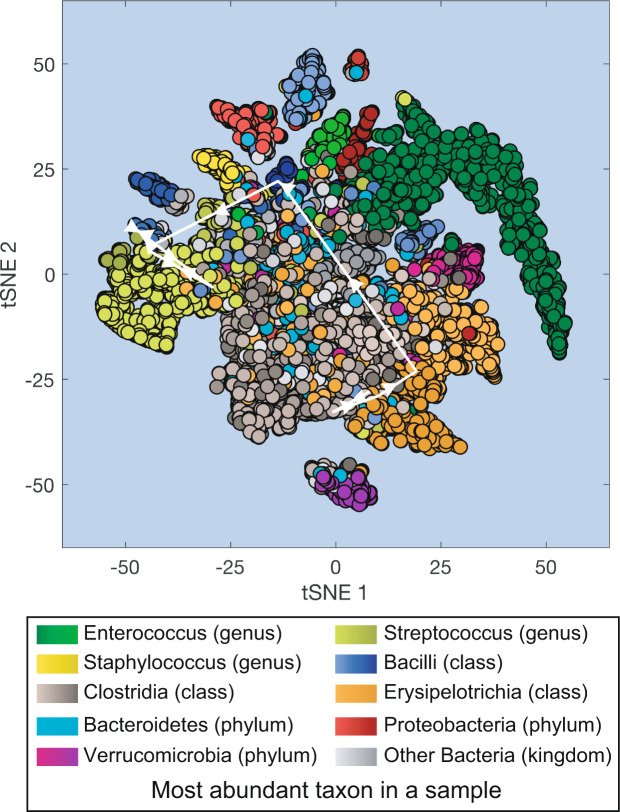
Projection of all microbiota samples from MSK patients receiving HCT onto a two-dimensional space using t-SNE (t-distributed stochastic neighbor embedding). White lines with arrows: microbiota compositional trajectory of the PatientID = 1511 also shown in Fig. 1.
  - ColorOrder: auxiliary integers used to group and sort the orders of ASVs in stacked barplot (Fig. 1)

The following data have been compiled as comma-separated value (csv) files in Figshare^35^. Folders “samples” and “taxonomy” contain a single file each at the moment. We expect to expand the contents of these folders as we compile more data (e.g., taxonomy of fungal ITS sequences, which is a recent interest of our team^36^) in the future. The files are the following:

Folder “counts”:

- tblcounts_asv_melt.csv: A melted table containing the sequence counts of each ASV detected in 12,546 stool samples
  - SampleID: stool sample identifier
  - ASV: identifier of the ASVs
  - Count: number of reads
- tblqpcr.csv: 16S rRNA qPCR data for 3,342 stool samples
  - SampleID: stool sample identifier
  - qPCR16S: 16S copies per gram of stool sample
- tblcounts_{asv,genus,family,order,class,phylum}_wide.csv: Wide-format taxa-by-sample table of counts at different taxonomic levels
  Folder “meta_data”:
- tblhctmeta.csv: The day and source of HCT for 1,278 patients
  - PatientID: deidentified identifier of patients
  - TimepointOfTransplant: deidentified day of allo-HCT (day of hematopoietic cell infusion). Out of 1,278 patients 1,212 have a single HCT, 64 have 2 HCTs and 2 patients have 3 HCTs.
  - HCTSource: hematopoietic cell sources for HCT patients (BM_unmodified: bone marrow; PBSC_unmodified: peripheral blood stem cells; TCD: T-cell depleted; cord: cord blood)
  - Disease: disease of patients
  - wbcPatientId: identifiers for the same patients included in a study of the role of the microbiota in white blood cell dynamics^3^
  - autoFmtPatientId: identifiers for the same patients included in our paper describing an autologous faecal microbiota trial^18^
  - nejmPatientId: whether the same patients from MSKCC samples were included in a recent multicentre study^15^
- tbldrug.csv: Timing and route of drug administration for 1,278 patients
  - PatientID: deidentified identifier of patients
  - StartTimepoint: deidentified day when drug administration started
  - StopTimepoint: deidentified day when drug administration stopped (including the day)
  - Factor: name of the drug
  - Category: category of the drug
  - AntiInfective: whether a drug is an anti-infective agent
  - Route: route of drug administration
  - StartDayRelativeToNearestHCT: start day of drug administration relative to the nearest day of bone marrow transplant
  - StopDayRelativeToNearestHCT: stop day of drug administration relative to the nearest day of bone marrow transplant
- tblInfectionsCidPapers.csv: The day of positive blood cultures for 426 patients and microbes (genera *Enterococcus*, *Escherichia*, *Klebsiella*, *Enterobacter*, *Pseudomonas*, *Stenotrophomonas*, and *Citrobacter*) analysed in previous publications from our team^13,23^
  - PatientID: deidentified identifier of patients
  - Timepoint: deidentified day of infection
  - InfectiousAgent: the bacteria causing infections
  - DayRelativeToNearestHCT: day of infection relative to the nearest day of bone marrow transplant
- tbltemperature.csv: temperatures for 1,249 patients
  - PatientID: deidentified identifier of patients
  - Timepoint: deidentified day when patient temperature was measured
  - MaxTemperature: Maximum temperature (unit: Fahrenheit) recorded on that day for that patient
  - DayRelativeToNearestHCT: day of temperature measurement relative to the nearest day of bone marrow transplant
- tblbc.csv: Daily measurements of white blood cells, platelets and red blood cells for 1,278 patients
  - PatientID: deidentified ID of patients
  - Day: deidentified day of blood cell measurement
  - BloodCellType: immature monocyte cells (ImmatureMonocytes), sezary cells (SezaryCells), variant lymphocyte cells (VariantLymphocytes), immature granulocyte cells (ImmatureGranulocytes), band cells (BandCells), basophil cells (Basophills), blast cells (BlastCells), eosinophil cells (Eosinophils), lymphocyte cells (Lymphocytes), neutrophil cells (Neutrophils), monocyte cells (Monocytes), platelet (Platelets), total white blood cells (WBCtotal), total red blood cells (RBCtotal)
  - Value: blood cell counts
  - Unit: K_per_μL (1,000 cells/μL) or M_per_μL (1,000,000 cells/μL)
  - DayRelativeToNearestHCT: day of blood cell measurement relative to the nearest day of bone marrow transplant
- tblVanA.csv: Results of PCR detection for *vanA* gene for 7,547 samples
  - SampleID: stool sample identifier
  - VanA: whether *vanA* gene is detected in the sample
  Folder “samples”:
- tblASVsamples.csv: The day of collection of 12,546 stool samples for 1,870 patients and the stool consistency
  - SampleID: stool sample identifier.
  - PatientID: deidentified identifier of patients
  - Timepoint: deidentified day of sample collection
  - Consistency: stool consistency
  - Accession: the NCBI SRA accession number for the most recent submission (among all duplicate submissions) of the same sequencing data corresponding to this sample
  - BioProject: project-level SRA identifier for the chosen ‘Accession’
  - DayRelativeToNearestHCT: day of sample collection relative to the nearest day of bone marrow transplant
  Folder “taxonomy”:
- tblASVtaxonomy_silva_v4v5_filter.csv: taxonomic information for 17,865 ASVs
  - ASV: identifier of the ASVs
  - Sequence: V4-V5 region of the 16S rRNA gene sequences
  - Kingdom, Phylum, Class, Order, Family, Genus: taxonomic classification
  - ConfidenceKingdom, ConfidencePhylum, ConfidenceClass, ConfidenceOrder, ConfidenceFamily, ConfidenceGenus: confidence assignment at each taxonomic level
  - HexColor: HEX color code used in microbiota composition bar plots (Fig. 1) and the t-SNE plot (Fig. 2).

## Technical Validation

### Displaying a patient timeline

We will display an individual patient’s microbiota timeline with associated clinical metadata as our first example analysis. We chose one patient (PatientID = 1511) and plotted the metadata by displaying the dates of sample acquisition relative to the HCT date (day −5 to day +21, Fig. 1). Therefore, the data filtering step here was straightforward: we excluded any sample that did not meet the criteria of PatientID = 1511 and −5≤DayRelativeToNearestHCT≤ + 21.

The top panel shows the maximum body temperature recorded for each day as a black dot, with red dots representing fevers (Temperature >37 °C or 100.4 °F). The second panel shows the anti-infectives given to the patient, with horizontal bars indicating daily administration of antibiotics, antivirals or antifungals. The third panel shows the count of neutrophils for the patient, with red dots representing neutropenia (<500 cell/μL). Finally, the bottom plot shows the gut microbiota composition analyzed by 16S rRNA amplicon sequencing in the 13 microbiota samples that fit the filtering criteria. Note that the values plotted are relative abundances. Relative abundances are obtained by dividing the number of read counts for that ASV by the number of total read counts in that sample (that total value may also be called the depth of that sample). Note that in the inference example below (Fig. 4) we will compute absolute abundances by multiplying the relative abundances by the qPCR16S column in tblqpcr.csv for that sample (the number of 16S copies per gram of stool sample measured by qPCR).

We can see how patient 1511 had a significant change in their microbiota composition after intravenous administration of piperacillin/tazobactam at day + 3 (indicated by a red dashed line). This combination of a beta-lactam (piperacillin) and a beta-lactamase inhibitor (tazobactam) is typically empirically administered at MSK to eradicate bacterial infections and, as such, was administered here in response to the fever occurring on that same day. We observe the expected drastic changes in the microbiota composition immediately following piperacillin/tazobactam administration^10^, with some bacteria dropping in their relative abundance (e.g., *Erysipelatoclostridium*) and other bacteria increasing (e.g., *Lactobacillus* and *Streptococcus*). The neutrophil counts show prolonged neutropenia (red), and engraftment on day 19 post-HCT.

### Visualizing the entire dataset of microbiota compositions

The large number of samples in our dataset provides a comprehensive overview of the microbiota compositional states experienced by the patients during their hospitalization. Here we used t-SNE^30^ to obtain a two-dimensional representation of all microbiota compositions which amount to 12,546 data points (Fig. 2). The t-SNE plot collapses high-dimensional microbiotas of HCT-receiving patients into several distinct clusters including high-diversity samples (located closer to the center of the plot) and lower diversity samples dominated by different bacteria. Samples dominated by *Enterococcus* (represented in green) are the most common type of low-diversity compositional state observed in the patients, and *Enterococcus* domination has been previously associated with higher risk of bloodstream infections by vancomycin-resistant *Enterococcus*^13^, with higher risk of graft-vs-host disease related death and a higher risk of overall mortality^20^.

We can overlay the trajectory of an individual patient’s microbiota composition on the t-SNE plot. This type of trajectories were used before to trace the success or failure of fecal microbiota transplants^18^. In this example we overlaid the trajectory of PatientID = 1511 between day −5 and day + 21 (white arrows in Fig. 2 connect the consecutive microbiota samples shown in Fig. 1).

### Inferring the impact of antibiotics on the microbiota using regularized regression

All patients in our dataset received antibiotics, but they received different antibiotics at different times and in different combinations. In previous studies we have used this variability to infer the impact of antibiotics on the different bacteria present in the gut microbiota by correlating microbiota changes with antibiotic exposures^3,10^.

**Fig. 3:**
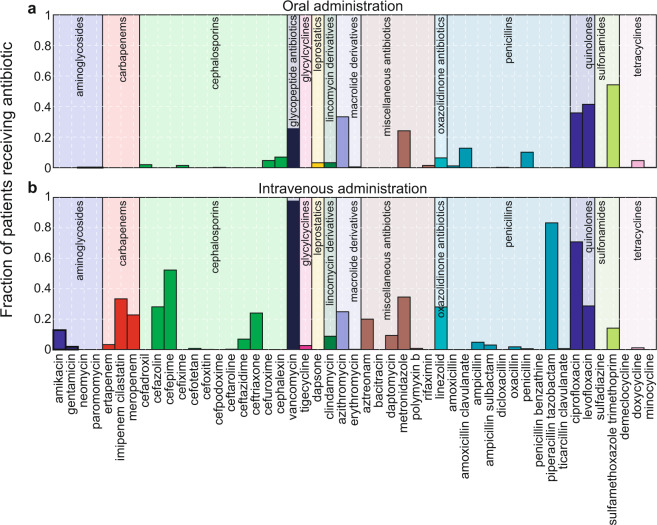
Relative frequency of antibiotics administered to HCT patients at MSK by oral (**a**) and intravenous (**b**) routes. Antibiotics are grouped by their categories and displayed in the same color within each group.

In this example we use a similar approach to infer the impact of antibiotics on the 20 most abundant microbes (ASVs) in the dataset. We selected for analysis only medications that belong to antibacterial classes, and we excluded atovaquone that is used for prophylaxis of pneumocystis and toxoplasma. As in a previous study from our team^10^, here we made a distinction between the administration route (e.g. oral or intravenous). Figure 3 shows the fraction of all patients who have ever received a specific antibiotic orally or intravenously (the two antibiotic administration routes accounts for 97% of all cases). The most frequently used antibiotics were sulfamethoxazole trimethoprim and vancomycin for oral and intravenous administrations respectively. Intravenous vancomycin was mainly used prophylactically for almost all patients; however, it was also empirically administered orally to kill infectious bacteria (e.g., *Clostridioides difficile*) in the gut because intravenous vancomycin may not reach adequate concentrations in the gastrointestinal tract. Quinolones (ciprofloxacin and levofloxacin), metronidazole, and azithromycin were given both orally and intravenously at similar frequencies among patients. Piperacillin/tazobactam was the second most commonly administered antibiotic by intravenous infusion.

For simplicity, we assume antibiotics independently affect the exponential growth rate of microbes and the magnitude of their effects can be inferred by the following linear regression:

1 $$\frac{d{\rm{\log }}{N}_{k}\left(t\right)}{dt}={g}_{k}+\mathop{\sum }\limits_{j=1}^{{M}_{a}}{\varepsilon }_{k,j}{u}_{j}\left(t\right)$$

where *N*_*k*_ is the absolute abundance of a microbial taxon *k* obtained by multiplying its relative abundance by the qPCR value, *g*_*k*_ is the intercept representing the maximum exponential growth rate in the absence of antibiotics, *ε*_*k,j*_ is the coefficient corresponding to antibiotic *j* representing the susceptibility of microbe *k*, *μ*_*j*_(*t*) is a binary variable that represents the presence/absence of the antibiotic *j* at time *t*, and *M*_*a*_ is the total number of antibiotics considered in the analysis. For any consecutive microbiota sample pair *p* observed between *t*_*p,i*_ (initial sample) and *t*_*p,f*_ (final sample), Eq. (1) can be transformed into an integral form

2 $${\rm{\log }}{N}_{k}\left({t}_{p,f}\right)-{\rm{\log }}{N}_{k}\left({t}_{p,i}\right)={g}_{k}\Delta {t}_{p}+\mathop{\sum }\limits_{j=1}^{{M}_{a}}{\varepsilon }_{k,j}{C}_{p,j}$$

where $\Delta {t}_{p}={t}_{p,f}-{t}_{p,i}$ represents the time interval between the two samples and ${C}_{p,j}={\int }_{{t}_{p,i}}^{{t}_{p,f}}{u}_{j}\left(t\right)dt$ represents the total exposure time to antibiotic *j* within the period. We discarded samples pairs when either or both of *N*_*k*_(*t*_*p,i*_) and *N*_*k*_(*t*_*p,f*_) is 0.

By taking all pairs of consecutive samples into account (*p* = 1, ···, *M*_*p*_), we formulated an independent linear regression problem for each microbe *i*

3 $$\left[\begin{array}{c}{\rm{\log }}\frac{{N}_{k}\left({t}_{1,f}\right)}{{N}_{k}\left({t}_{1,i}\right)}\\ \begin{array}{c}\vdots \\ {\rm{\log }}\frac{{N}_{k}\left({t}_{p,f}\right)}{{N}_{k}\left({t}_{p,i}\right)}\\ \vdots \end{array}\\ {\rm{\log }}\frac{{N}_{k}\left({t}_{{M}_{p},f}\right)}{{N}_{k}\left({t}_{{M}_{p},i}\right)}\end{array}\right]=\left[\begin{array}{c}\begin{array}{ccc}\begin{array}{cc}\Delta {t}_{1} & {C}_{1,1}\end{array} & \begin{array}{ccc}\ldots  & {C}_{1,j} & \ldots \end{array} & {C}_{1,{M}_{a}}\end{array}\\ \begin{array}{c}\vdots \\ \begin{array}{ccc}\begin{array}{cc}\Delta {t}_{p} & {C}_{p,1}\end{array} & \begin{array}{ccc}\ldots  & {C}_{p,j} & \ldots \end{array} & {C}_{p,{M}_{a}}\end{array}\\ \vdots \end{array}\\ \begin{array}{ccc}\begin{array}{cc}\Delta {t}_{{M}_{p}} & {C}_{{M}_{p},1}\end{array} & \begin{array}{ccc}\ldots  & {C}_{{M}_{p},j} & \ldots \end{array} & {C}_{{M}_{p},{M}_{a}}\end{array}\end{array}\right]\left[\begin{array}{c}\begin{array}{c}{g}_{k}\\ {\varepsilon }_{k,1}\end{array}\\ \begin{array}{c}\vdots \\ {\varepsilon }_{k,j}\\ \vdots \end{array}\\ {\varepsilon }_{k,{M}_{a}}\end{array}\right]$$

Equation (3) can be solved by penalized linear regression to avoid overfitting. Using simplified notations for Eq. (3), i.e., **Y**_*k*_ = **D**_*k*_**X**_*k*_, we solved the following ridge regression for each given penalty parameter *λ*

4 $${\left({{\bf{X}}}_{k}\right)}_{{\rm{\lambda }}}^{{\rm{opt}}}=\mathop{{\rm{argmin}}}\limits_{{{\bf{X}}}_{k}}\left({\left\Vert {{\bf{Y}}}_{k}-{{\bf{D}}}_{k}{{\bf{X}}}_{k}\right\Vert }_{F}^{2}+{\rm{\lambda }}{\left\Vert {{\boldsymbol{X}}}_{k}\right\Vert }_{F}^{2}\right)$$

where $| | \,\cdot \,| {| }_{F}^{2}$ is the Frobenius norm. We chose *λ* to minimize the sum of squares error on unseen (test) data using 3-fold cross-validation (with the Matlab option of 10 Monte-Carlo repetitions) and the optimal *λ* was then applied to the entire dataset for estimating the values of growth and susceptibility parameters.

We limited ourselves to sample pairs that are at most 3 days apart (the minimum interval that includes >50% data) and both have absolute abundances determined by qPCR data. We also focused on the 20 most abundant ASVs that have non-zero abundances among samples—from a total of 289 patients—fulfilling the minima criteria for inclusion in the regression. We included antibiotics administrated orally and intravenously but separated our analysis for the two administration routes. We also included only anti-bacterial drugs and grouped these drugs based on their drug category (Fig. 3). We separated metronidazole and aztreonam from the miscellaneous class as independent groups and removed other drugs in that class. For most anti-bacterial antibiotic classes, it is typical that one or two antibiotics were administered much more often than the rest drugs in the same class (Fig. 3).

**Fig. 4:**
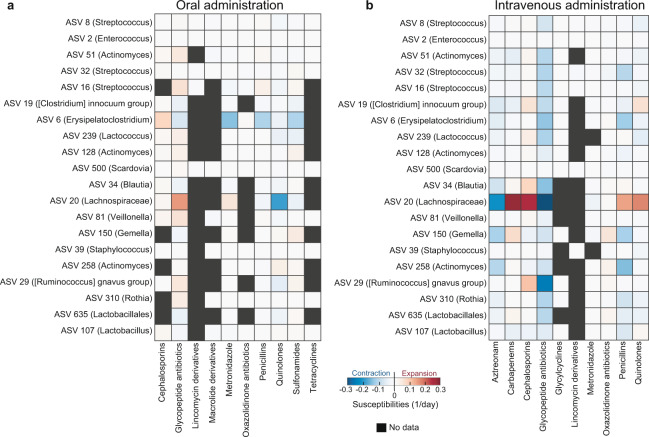
Effects of orally (**a**) and intravenously (**b**) administered antibiotics on microbes. Each ASV was labeled by its lowest taxonomy level that is not unclassified.

The effect sizes obtained using this model show that the route of administration influences the effects of antibiotics on microbiota compositions (Fig. 4): intravenous drugs tend to be more effective to reduce microbial abundances in the gut relative to oral drugs. Piperacillin/tazobactam (penicillins) and vancomycin (glycopeptide antibiotics)—the most commonly administered antibiotics by intravenous infusion—have the strongest inhibitory effects on many commensal bacteria such as *Blautia*, *Ruminococcus*, *Erysipelatoclostridium*. The timeline of microbial composition shift in Fig. 1 illustrated the inferred negative effect of piperacillin/tazobactam on ASV 6 (*Erysipelatoclostridium*) at day 3. However, the expansion of *Lactobacillus* upon administration of piperacillin/tazobactam—suggesting a positive antibiotic effect—was incorrectly inferred. This potential caveat due to the simplicity of our model is discussed below with details.

### Intestinal domination increases a patient’s risk of bloodstream infection

**Fig. 5:**
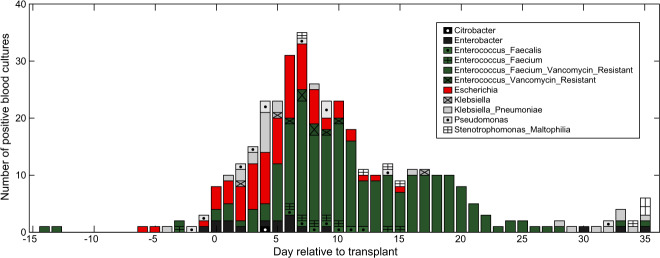
A compilation of cases of positive blood culture infections for the bacteria analyzed in previous publications^13,23^. Here the period ranging from day −15 to day + 35 around the day of HCT (day 0) is shown, and we highlight the cases of *Enterococcus* (in green) and *Escherichia* (in red) analyzed below in Fig. 6.

Patients undergoing allo-HCT are at high risk to bloodstream infections, especially during neutropenia (Fig. 5). We previously reported patient risk to bloodstream infection by *Enterococcus* after the gut microbiota became dominated by *Enterococcus*, where domination was defined as a relative abundance >30%^13^. A similar analysis was later conducted to determine the risk that a patient would develop a bloodstream infection by various gram-negative bacteria after the gut microbiota became dominated by a bacteria of the same genus, with domination again defined as >30%^23^.

**Fig. 6:**
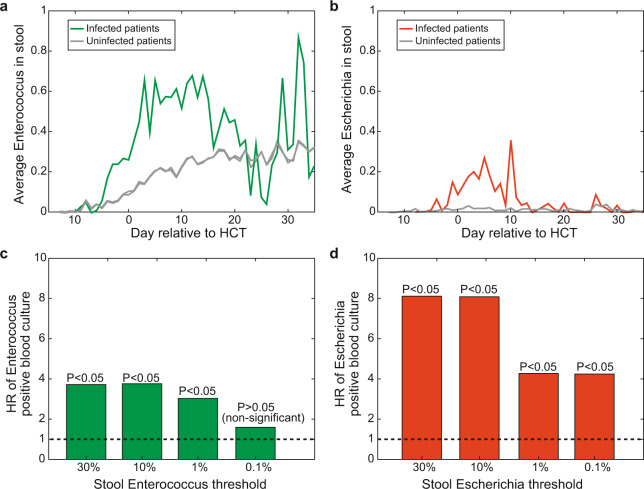
(**a**) The average abundance of *Enterococcus* is higher in patients who got Enterococcal bloodstream infections (n = 79) than in patients who did not (n = 940), especially in the critical period of two weeks after the transplant (day 0, where ‘Day’ is relative to the nearest allo-HCT transplant). (**b**) The average abundance of bacteria of the genus *Escherichia* is higher in patients who got a bloodstream infection by that genus (n = 52) than in patients who did not (n = 967), especially in the critical period of two weeks after the transplant (day 0). (**c**) The hazard ratio calculated for the risk of bloodstream infection after the patient was detected with an intestinal domination. These analyses were previously done by defining intestinal domination at an abundance threshold of 30% domination^13,23^. The results shown here reveal that domination redefined at an abundance threshold as small as 1% still increases the risk of bloodstream infection by *Enterococcus*. (**d**) The presence in the stool is even a stronger predictor of bloodstream infection for the case *Escherichia*, for which even levels of 0.1% have a significant association.

Here we run that same type of survival analysis: We restricted our analysis to the period from day −15 to day +35 and we determined the risk of a bloodstream infection with *Enterococcus* (Fig. 6a) and *Escherichia* (Fig. 6b). We used the Cox proportional hazards model with domination as a time-dependent covariate, which starts at value 0 and changes to 1 once the relative abundance of the genus in the stool increases above a given threshold. We first conducted analysis assuming a 30% domination value. Recapitulating our previous results, intestinal domination by *Enterococcus* had a hazard ratio of 3.8 for blood-stream infection by *Enterococcus* (P < 0.05, with a [2.6–5.5] 95% confidence interval) and intestinal domination by *Escherichia* had a hazard ratio of 8.1 for bloodstream infection by *Escherichia* (P < 0.05 with a [4.4–15.0] 95% confidence interval).

We then repeated the same analysis for dominations defined at 10%, 1% and 0.1% (Fig. 6c,d). The analyses reveal that *Enterococcus* abundances as small as 1% increase the risk of blood-stream infection by *Enterococcus* significantly (P < 0.05). The case for *Escherichia* was more sensitive: *Escherichia* abundances as small as 0.1% increased the risk of bloodstream infection by *Escherichia* significantly (P < 0.05). Such analyses, all conducted using the Cox proportional hazards model, can be extended to find other factors that increase the risk of bloodstream infections and other clinical complications of allo-HCT.

### Discussions and potential caveats

We present repositories for a longitudinal microbiota dataset obtained from efforts at MSK from the past decade of monitoring cancer patients hospitalized to receive allo-HCT. These microbiota data, combined with the curated clinical metadata presented here, can serve as powerful hypothesis generators for microbiome studies. For example, we illustrate the use of these data with five analyses and we provide links to code repositories where these examples can be followed by the interested user. Despite the comprehensiveness of our data collection, not all factors (e.g., diet) that influence gut microbiota composition have been included and it is not yet available to analyze their effects using our datasets. We expect to release more data types in the future.

We showed an example of inference where we quantified the impact of antibiotics on the 20 most abundant microbes (ASVs). That example (Fig. 4) illustrates how to pose a supervised question to address an important microbiota problem: how different antibiotics might have an impact on the different bacteria that makeup the microbiota. We had addressed a related question before using straightforward statistical tests for a dataset of 94 patients, where we determined whether an antibiotic could lead to intestinal domination^13^. We have also used more sophisticated Bayesian methods to infer the impacts of antibiotics on the microbiota using a dataset with 18 of these patients^10^. In the present manuscript, we presented a similar approach and used a simple model of exponential bacterial growth combined with penalized least squares regression to solve the same problem for the most abundant taxa, but now for a dataset with a much larger number of patients. Despite the simpler linear model, the results captured the negative impact of piperacillin/tazobactam on many commensal bacteria, confirming our previous studies^3,10^. We here also visualized *Lactobacillus* expansion and intestinal domination, illustrated in Fig. 1, but this was not captured by our model. The most likely reason that our simple model of exponential growth could not capture this is that after an expansion of *Lactobacillus* following the administration of piperacillin/tazobactam, its abundance remains relatively unchanged over multiple consecutive days. Since the antibiotics continued to be administered during the time, the inference will combine all the data and conclude that—on average—there is no measurable impact of piperacillin/tazobactam on *Lactobacillus*. Therefore, the model as developed here is only sensitive to transient expansions of bacteria but unable to capture bacterial expansions followed by sustained dominations. One possible way to resolve this issue in future iterations of the analysis is to include more realistic details in a more complicated model, such as an ecological carrying capacity and microbial interactions, as done before^3,10,37–39^.

Finally, some notes of caution on generalizing these findings: The patient cohort presented here consists entirely of patients undergoing allo-HCT at MSK, which represent states of the human immune system and microbiota compositions far from that of healthy people. The microbiota changes observed during a patient’s treatment occur in parallel with a high burden of antibiotic exposure, dietary perturbation, and gastrointestinal inflammation induced by the conditioning regimen. The combination of these profound microbiota perturbations rarely occurs for healthy individuals. The clinical complications associated with the microbiota changes observed in these patients may also be specific. For example, we found that *Enterococcus* or *Escherichia* expansions increase the risk of bloodstream infections by those bacteria when detected in the microbiota at abundances as low as 1% (Fig. 6). This risk may be specific to transplant recipients damaged by conditioning regimen with injury to the intestinal barrier that could facilitate the translocation of pathogens from the gut into the blood^40^. Still, allo-HCT patients provide a unique opportunity to study microbiota dynamics in such extreme situations. There are many other opportunities for analysis that we did not discuss here such as the impact of the microbiota on the counts of immune cells in circulation^3^. The findings made from these data may be applicable to other HCT patients and perhaps other patients undergoing similar perturbations.

## Data Availability

The customized Matlab code (Matlab 2018a) used for the examples provided below is available in the GitHub repository (https://github.com/liaochen1988/MSKCC_Microbiome_SD2021_Scripts) with each part in a separate directory: • Figure 1: example 1_display_patient_timeline/main.m • Figure 2: example 2_visualize_compositional_states/main.m • Figure 3: example 3_drug_administration_stats/main.m • Figure 4: example 4_impacts_of_antibiotics/main.m • Figures 5, 6: example 5_survival_analysis/main.m
