# Author Correction: Compilation of longitudinal microbiota data and hospitalome from hematopoietic cell transplantation patients

**DOI:** 10.1038/s41597-021-00903-0

**Published:** 2021-04-23

**Authors:** Chen Liao, Bradford P. Taylor, Camilla Ceccarani, Emily Fontana, Luigi A. Amoretti, Roberta J. Wright, Antonio L. C. Gomes, Jonathan U. Peled, Ying Taur, Miguel-Angel Perales, Marcel R. M. van den Brink, Eric Littmann, Eric G. Pamer, Jonas Schluter, Joao B. Xavier

**Affiliations:** 1 Program for Computational and Systems Biology, Memorial Sloan-Kettering Cancer Center, New York, NY USA; 2 Department of Health Sciences, Università degli Studi di Milano, Milan, Italy; 3 Infectious Disease Service, Department of Medicine, and Immunology Program, Sloan Kettering Institute, New York, NY USA; 4 Department of Immunology, Memorial Sloan-Kettering Cancer Center, New York, NY USA; 5 Adult Bone Marrow Transplantation Service, Department of Medicine, Memorial Sloan Kettering Cancer Center, New York, NY USA; 6 Weill Cornell Medical College, New York, NY USA; 7 Duchossois Family Institute, University of Chicago, Chicago, IL USA

**Keywords:** Cancer, Databases, Time series, Microbiome, Data mining

Correction to: *Scientific Data* 10.1038/s41597-021-00860-8, published online 02 March 2021

The original version of this Data Descriptor omitted the following author from the Author List: Ying Taur. The URL in reference 35 has also been corrected to 10.6084/m9.figshare.c.5271128. These errors have now been corrected in both the PDF and HTML versions of the Article.

